# Influence of Sex, BMI, and Skin Color on the Accuracy of Non-Invasive Cuffless Photoplethysmography-Based Blood Pressure Measurements

**DOI:** 10.3389/fphys.2022.911544

**Published:** 2022-06-29

**Authors:** Dean Nachman, Arik Eisenkraft, Nir Goldstein, Arik Ben-Ishay, Meir Fons, Roei Merin, Yftach Gepner

**Affiliations:** ^1^ Heart Institute, Hadassah Ein Kerem Medical Center, Jerusalem, Israel; ^2^ Institute for Research in Military Medicine, Faculty of Medicine, The Hebrew University of Jerusalem and the Israel Defense Force Medical Corps, Jerusalem, Israel; ^3^ Biobeat Technologies LTD., Petach Tikva, Israel; ^4^ Department of Epidemiology and Preventive Medicine, School of Public Health, Sackler Faculty of Medicine and Sylvan Adams Sports Institute, Tel Aviv University, Tel-Aviv, Israel

**Keywords:** blood pressure, photoplethysmography, age, BMI, sex

## Abstract

Vital signs obtained by photoplethysmography-based devices might be influenced by subcutaneous fat and skin color. This observational comparison study aimed to test the accuracy of blood pressure (BP) measurements between a photoplethysmography-based device and cuff-based BP device in ambulatory individuals, coming for a routine BP checkup. Systolic BP (SBP) and diastolic BP (DBP) measurements were stratified based on sex, BMI (<25; 25 ≤BMI<30; 30 ≤kg/m^2^), and skin color (types 1–3 and 4–6 by the Fitzpatrick scale). A total of 1548 measurements were analyzed. Correlations of SBP and DBP between the devices among males/females were between 0.914–0.987 (*p* < 0.001), and Bland-Altman analysis showed a bias of less than 0.5 mmHg for both sexes. Correlations of SBP and DBP between the devices among BMI groups were between 0.931–0.991 (*p* < 0.001), and Bland-Altman analysis showed a bias of less than 1 mmHg for all. Correlations of SBP and DBP between the devices among the skin color groups were between 0.936–0.983 (*p* < 0.001), and Bland-Altman analysis showed a bias of less than 1 mmHg for all. This study shows similar and high agreements between BP measurements obtained using a PPG-based non-invasive cuffless BP device and a cuff-based BP device across sex, BMI, and skin color groups.

## Introduction

Collecting physiological vital signs, such as blood pressure and blood oxygen saturation, is regarded as a basic component in the clinical assessment of individuals. One of the challenges in collecting these vital signs is the accuracy of the measurements in individuals that are overweight or obese and individuals with dark skin color, as measurements seem to be less reliable (Ries et al., 1985; Ries et al., 1989; Jubran and Tobin, 1990; Zeballos and Weisman, 1991; Young, 1995; O’Brien, 1996; Palatini and Parati, 2011; Palatini et al., 2019). This is still an ongoing debate with conflicting evidence and is especially relevant in devices employing photoplethysmography (PPG) technology. As the US population is comprised of about 13.4% African-Americans (see https://www.census.gov/), and 42.4% of the US population is obese (see https://www.cdc.gov/obesity/data/adult.html), verifying the accuracy of such devices in these sub-populations is highly important to allow their general use. Moreover, though sex is regarded as a fundamental aspect of human physiology, it is usually not considered in the design of studies, or in developing personalized medical strategies (Miller, 2014).

In the medical literature, several research groups have found that skin pigmentation does not affect the bias or precision of pulse oximetry, and has no clinically significant effect on pulse oximetry signal quality (Bothma et al., 1996; Adler et al., 1998; Pipek et al., 2021). In a recent study in patients with COVID-19 pneumonitis, measurement of SpO_2_ was not affected by patient ethnicity to a clinically significant degree (Wiles et al., 2021). Other research groups have found that several pulse oximeters overestimated arterial oxygen saturation during hypoxia in dark-skinned individuals (Bickler et al., 2005; Feiner et al., 2007); in a study of two large cohorts, dark-skinned patients had nearly three times the frequency of occult hypoxemia that was not detected by pulse oximetry as White patients (Sjoding et al., 2020). According to The US Food and Drug Administration’s standards for studies undertaken to test the accuracy of pulse oximeters, they should include among other components a range of skin pigmentations, including at least two darkly pigmented subjects or 15% of the study population, whichever is larger (US Food and Drug Administration, 2013).

In recent years, exciting algorithm and hardware improvements in PPG technology allowed the development and validation of non-invasive monitoring methods of blood pressure and other advanced hemodynamic parameters (Nachman et al., 2020a; Nachman et al., 2020b; Nachman et al., 2021). Whether these devices provide accurate measurements of such parameters in both sexes, in various skin tones, and body girth was not fully studied yet. In the current study, we aimed to test the accuracy of blood pressure (BP) measurements using a novel PPG-based device stratifying the data based on sex, BMI, and skin color, in a field setting.

## Methods

### Study Population

This was an observational comparative field study. Male and female volunteers of all ages were recruited as they arrived for a BP checkup provided by skilled evaluators from the Israeli National Emergency Medical Services (EMS) in designated BP measurement public screening stations deployed across the country. Each participant has signed an informed consent form as defined by the Institutional Review Board of the Tel-Aviv Medical Center, Tel-Aviv, Israel (0032-15-TLV).

### Study Protocol

Reference calibration BP measurements using a cuff-based device (Welch Allyn DuraShock DS65 hand sphygmomanometer, Skaneateles Falls, NY 13153, United States) were taken from each participant and used as a baseline calibration value for the PPG-based wearable device. Next, the wearable device was attached to each of the subjects by the EMS personnel and left for 30 s to acquire a PPG signal reading while the volunteers were at rest. This was repeated on the other hand to show there are no differences. After obtaining a signal, BP was measured concomitantly in all subjects using both devices - the standard cuff-based device previously mentioned on one hand and the PPG-based device on the opposite arm. Since the cuff-based device occludes blood flow to the arm, PPG measurements cannot be obtained if the device is located on that same arm. When comparing the PPG-based device to the cuff-based BP device, and since it takes time between the systolic and the diastolic measurements when using the cuff-based device, the systolic value of the PPG-based device was recorded once the systolic value was reached in the cuff-based device, and once the diastolic value was reached in the cuff-based device, the diastolic measurement in the PPG-based device was recorded. Adverse events were recorded by the research team. The evaluators were also recording basic demographic details of each participant, including height, weight, and based on the Fitzpatrick color scale provided to each, also determined the skin color type of each participant. There was one evaluator per patient, as the aim was to sustain the local routine blood pressure check-up protocol.

### The PPG-Based Device

The skin-attached device (BB-613WP, Biobeat Technologies Ltd., Petah Tikva, Israel) is indicated for use in measuring and displaying pulse oxymetry and blood pressure values, as well as other advanced cardio-pulmonary parameters, using reflective PPG. Following transmission of several specific wavelengths of light, a detector measures the changing absorbance resulting from the pulsating arterial blood at each of the wavelengths. When using the device, a preliminary calibration step is performed using an FDA-cleared cuff-based BP device. The calibration value is entered into a user’s application, valid for up to 3 months (relevant supportive clinical data has been presented by the company and accepted by the FDA), after which a new calibration measurement should be taken and introduced into the application using the same method. From that moment on, the PPG measurements are analyzed using pulse wave transit time (PWTT).

### Statistical Analysis

Participants were stratified by sex, by BMI (BMI<25, defined as normal weight; 25 ≤BMI<30, defined as overweight; and 30 ≤BMI, defined as obese; based on the World Health Organization (WHO) definitions, https://www.who.int/news-room/fact-sheets/detail/obesity-and-overweight), and skin color based on the Fitzpatrick scale, divided between type 1–3 and type 4–6 (Fitzpatrick, 1988) (stratifications based on USFDA, 2013). Correlation analysis was performed using Pearson’s correlation, and agreement was evaluated based on the Bland-Altman method using 95% limits of agreement (LOA). *p*-values were set at 0.05. Data analysis was performed using GraphPad Prism 8 and presented as mean ± standard deviation.

## Results

Table 1 includes demographic data and characteristics of the participants, stratified by BMI and skin color. No adverse events were recorded during the study. A total of 1548 samples recorded from 1057 participants were included in the analysis. For SBP, the Bland-Altman analysis of the whole group showed that the PPG had a bias of -0.02 ± 3.7 mmHg with 7.3 and −7.2 mmHg 95% LOA, and Pearson’s correlation (r) of 0.985. For DBP, the Bland-Altman analysis of the whole group showed that the PPG had a bias of −0.3 ± 4.2 mmHg with 8.0 and −8.6 mmHg 95% LOA, r = 0.931.

**TABLE 1:** Demographic data of the participants.

Characteristic	Mean ± SD		
Age (years)	35.1 ± 23.8		
Sex (M/F)	592/467		
BMI (kg/m^2^)	24.1 ± 4.7		
Fitzpatrick	3.3 ± 1.5		
Total samples			
	n (%)	Males (%)	Age (years)
	1548 (100%)	664 (42.9%)	35.1 ± 23.8
BMI			
BMI<25	1057 (68.3%)	417 (39.4%)	29.1 ± 21.4
25 ≤ BMI<30	346 (22.4%)	168 (48.4%)	43.8 ± 24.3
30 ≤ BMI	145 (9.4%)	79 (54.5%)	48.6 ± 23.5
Skin color type			
Fitzpatrick 1–3	936 (60.5%)	420 (44.9%)	35.2 ± 23.8
Fitzpatrick 4–6	612 (39.5%)	244 (39.9%)	34.9 ± 23.8

Body mass index (BMI): overweight defined as 25 ≤BMI <30, and obese defined as 30 ≤BMI. The Fitzpatrick color scale: Type 1—always burns, never tans, palest, can have freckles; Type 2—usually burns, tans minimally, light-colored but darker than fair; Type 3—sometimes mild burn, tans uniformly, golden honey or olive; Type 4—burns minimally, always tans well, moderate brown; Type 5—very rarely burns, tans very easily, dark brown; and Type 6—never burns, deeply pigmented dark brown to darkest brown.

**TABLE 2:** Level of agreement between the PPG-based wearable measurements across commonly accepted standard.

	<5 mmHg		5–7 mmHg		7–10 mmHg		>10 mmHg	
	SBP	DBP	SBP	DBP	SBP	DBP	SBP	DBP
Sex								
Male	79.2%	80.1%	15.9%	13.7%	4.9%	6.2%	0.0%	0.0%
Female	76.9%	80.0%	17.1%	14.7%	5.6%	4.9%	0.3%	0.3%
Weight category								
Normal weight	77.0%	79.7%	17.7%	14.9%	5.0%	5.3%	0.3%	0.2%
Overweight	80.7%	79.6%	14.2%	13.9%	5.1%	6.2%	0.0%	0.4%
Obese	76.3%	83.1%	16.1%	11.9%	7.6%	5.1%	0.0%	0.0%
Skin color								
Fitzpatrick 1–3	77.3%	80.8%	17.4%	13.9%	5.3%	5.1%	0.0%	0.2%
Fitzpatrick 1–3	78.8%	78.8%	15.4%	14.9%	5.3%	6.0%	0.5%	0.2%

Number in the table are percentage of measurements with a delta of less than 5, 5–7, 7–10 or above 10 mmHg between the reference device and the PPG-based wearable measurements. Body mass index (BMI): overweight defined as 25 ≤BMI <30, and obese defined as 30 ≤BMI. The Fitzpatrick color scale: Type 1—always burns, never tans, palest, can have freckles; Type 2—usually burns, tans minimally, light-colored but darker than fair; Type 3—sometimes mild burn, tans uniformly, golden honey or olive; Type 4—burns minimally, always tans well, moderate brown; Type 5—very rarely burns, tans very easily, dark brown; and Type 6—never burns, deeply pigmented dark brown to darkest brown.

**FIGURE 1:**
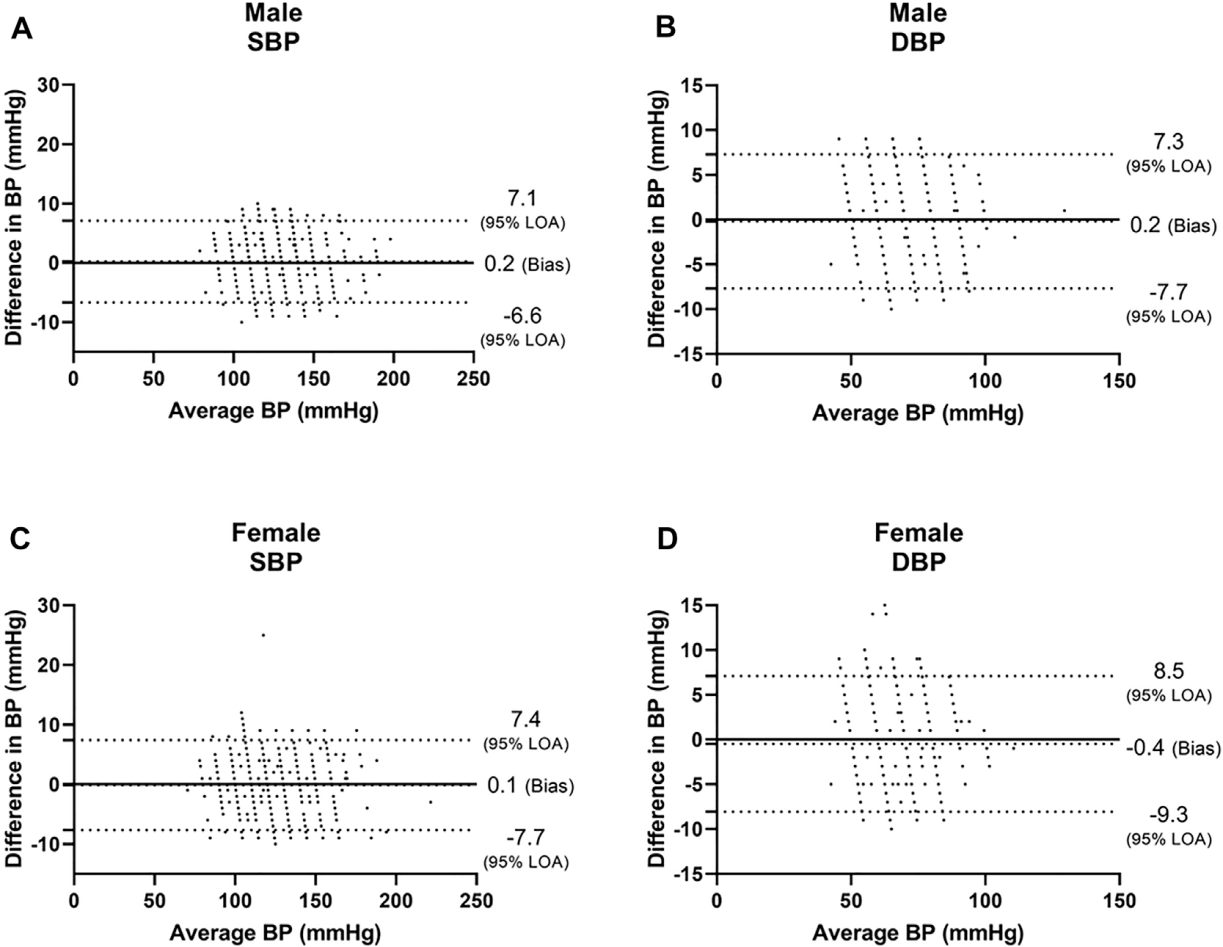
Bland-Altman analysis of males and females. SBP systolic blood pressure, DBP diastolic blood pressure.

**FIGURE 2:**
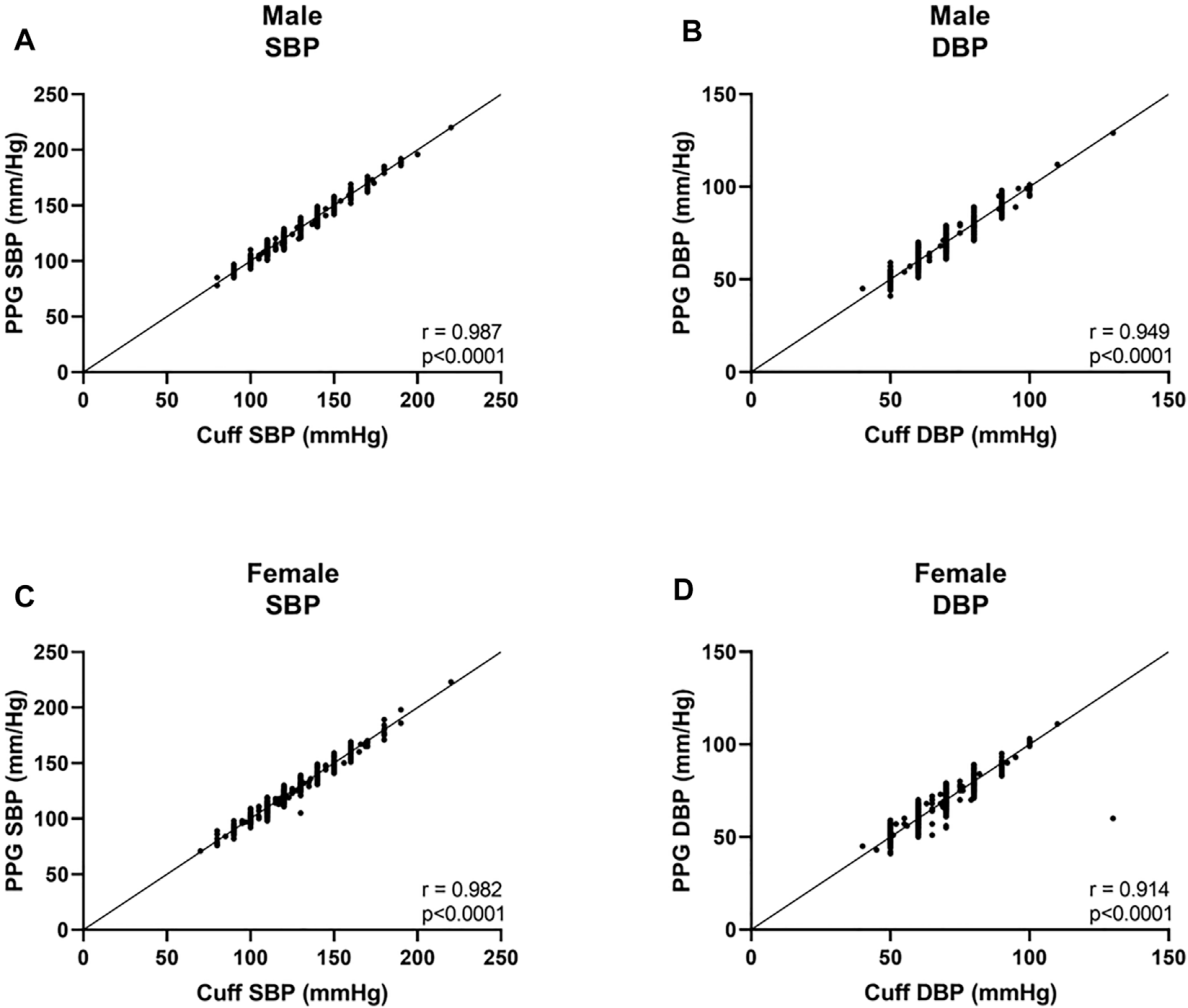
Correlation curves of males and females. SBP systolic blood pressure, DBP, diastolic blood pressure.

Among males, we found a bias of 0.2 ± 3.5 mmHg with 7.1 and −6.6 mmHg 95% LOA for SBP, with r = 0.987 (Figures 1A, 2A), and a bias of 0.2 ± 3.8 mmHg with 7.3 and −7.7 mmHg 95% LOA for DBP, with r = 0.949 (Figures 1B, 2B). Among females, we found a bias of 0.1 ± 3.8 mmHg with 7.4 and −7.7 mmHg 95% LOA for SBP, with r = 0.982 (Figures 1C, 2C), and a bias of −0.4 ± 3.9 mmHg with 8.5 and −9.3 mmHg 95% LOA for DBP, with r = 0.914 (Figures 1D, 2D).

**FIGURE 3:**
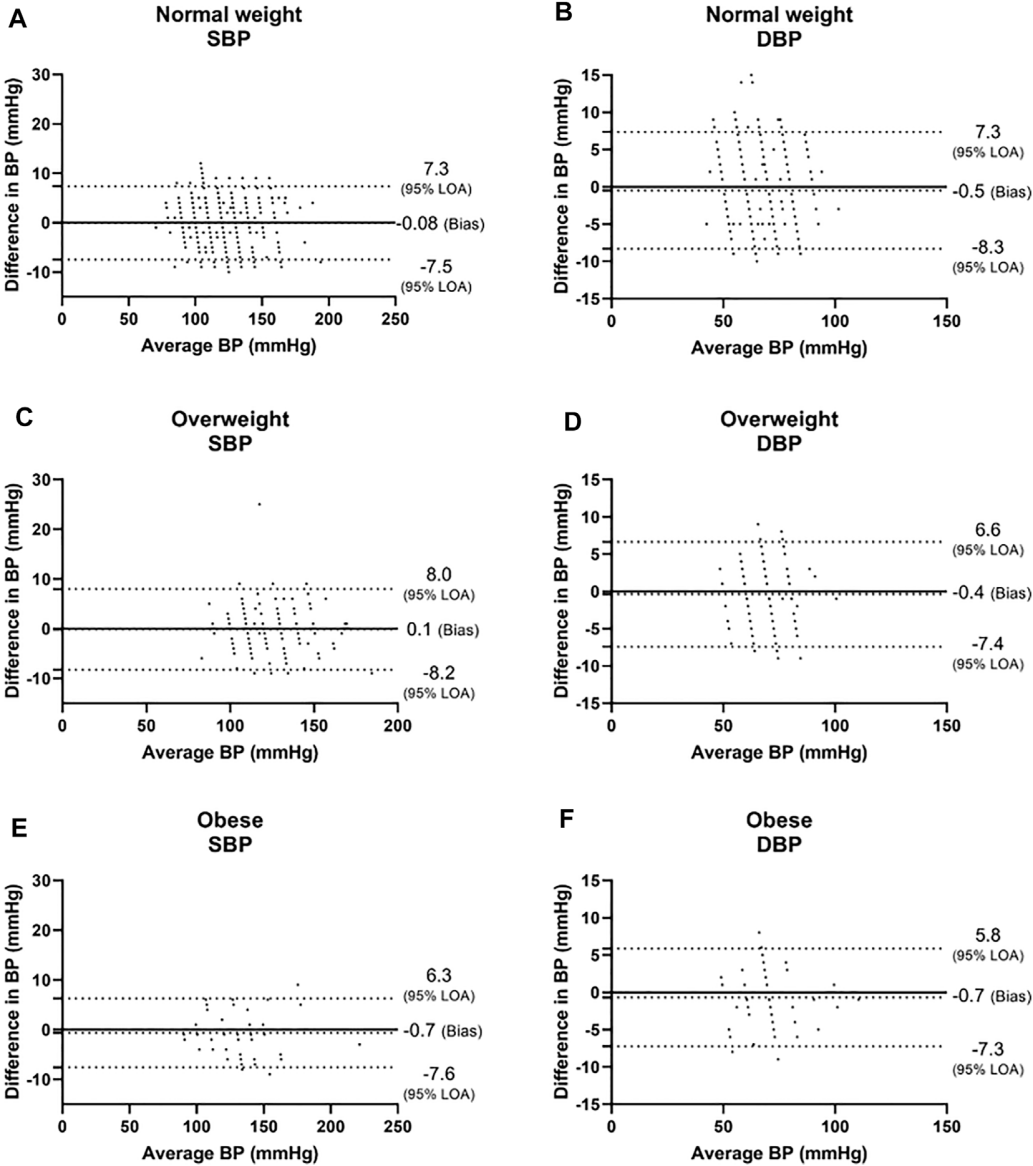
Bland-Altman analysis of the body mass index (BMI) groups. Normal weight was defined as BMI<25, overweight was defined as 25 ≤BMI <30, and obese was defined as 30 ≤BMI. LOA level of agreement, SBP systolic blood pressure, DBP diastolic blood pressure.

**FIGURE 4:**
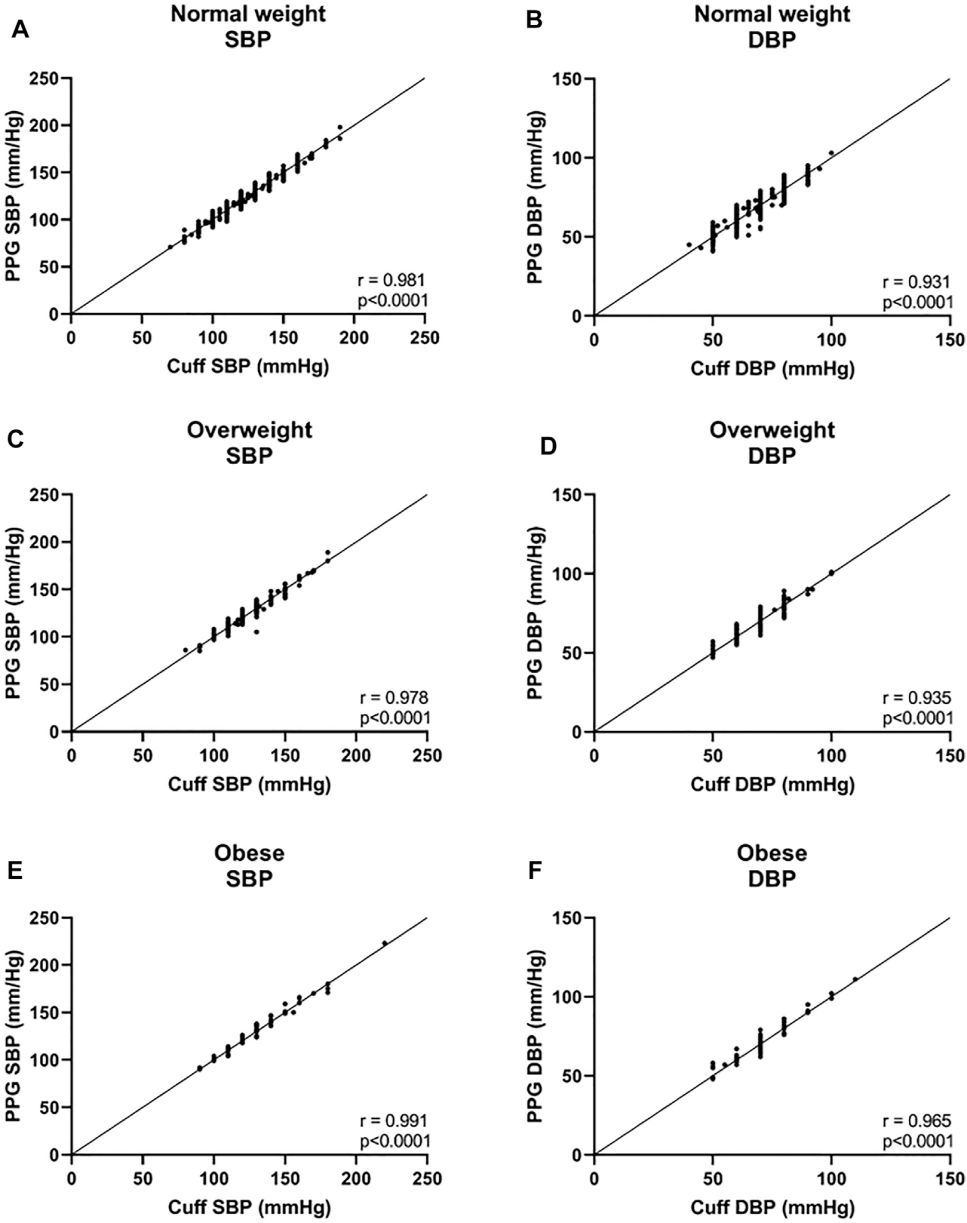
Correlation curves of the body mass index (BMI) groups. Normal weight was defined as BMI <25, overweight was defined as 25 ≤BMI <30, and obese was defined as 30 ≤BMI. SBP systolic blood pressure, DBP diastolic blood pressure.

In the three BMI groups, for SBP, a bias of −0.08 ± 3.8 mmHg with 7.3 and −7.5 mmHg 95% LOA was found in the normal weight group, r = 0.981; 0.1 ± 4.1 mmHg with 8.0 and −8.2 mmHg 95% LOA in the overweight group, r = 0.978; and −0.7 ± 3.5 mmHg with 6.3 and −7.6 mmHg 95% LOA in the obese group, r = 0.991 (Figures 3A,C,E, and Figures 4A,C,E). For DBP, a bias of −0.5 ± 4.0 mmHg with 7.3 and −8.3 mmHg 95% LOA in the normal weight group, r = 0.931; −0.4 ± 3.6 mmHg with 6.6 and −7.4 mmHg 95% LOA in the overweight group, r = 0.935; and −0.7 ± 3.3 mmHg with 5.8 and −7.3 mmHg 95% LOA in the obese group, r = 0.965 (Figures 3B,D,F, and Figures 4B,D,F).

**FIGURE 5:**
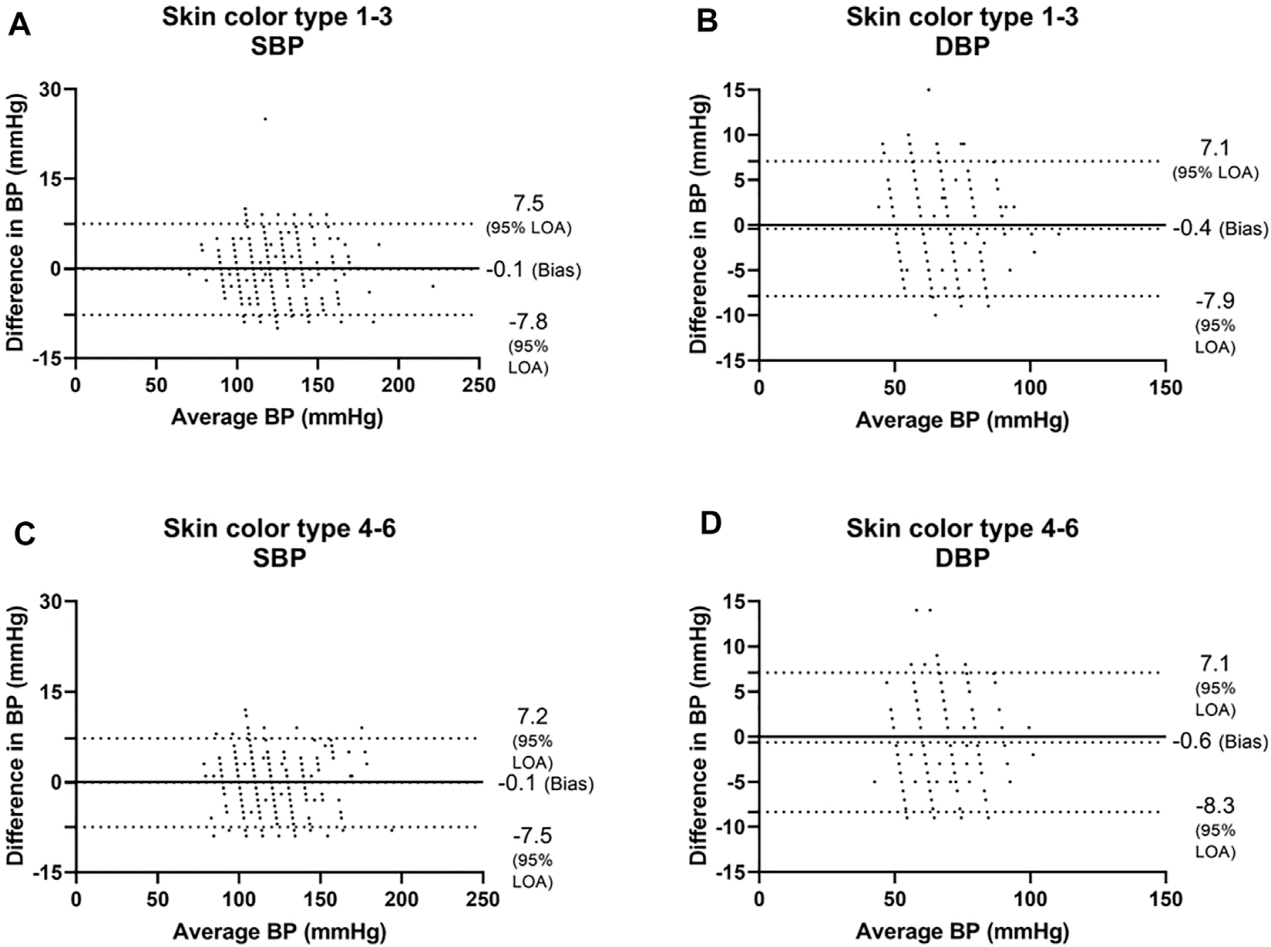
Bland-Altman analysis of the body’s skin color is based on the Fitzpatrick color scale (Fitzpatrick, 1988). Type 1—always burns, never tans, palest, can have freckles; Type 2—usually burns, tans minimally, light-colored but darker than fair; Type 3—sometimes mild burn, tans uniformly, golden honey or olive; Type 4—burns minimally, always tans well, moderate brown; Type 5—very rarely burns, tans very easily, dark brown; and Type 6—never burns, deeply pigmented dark brown to darkest brown. SBP systolic blood pressure, DBP diastolic blood pressure.

**FIGURE 6:**
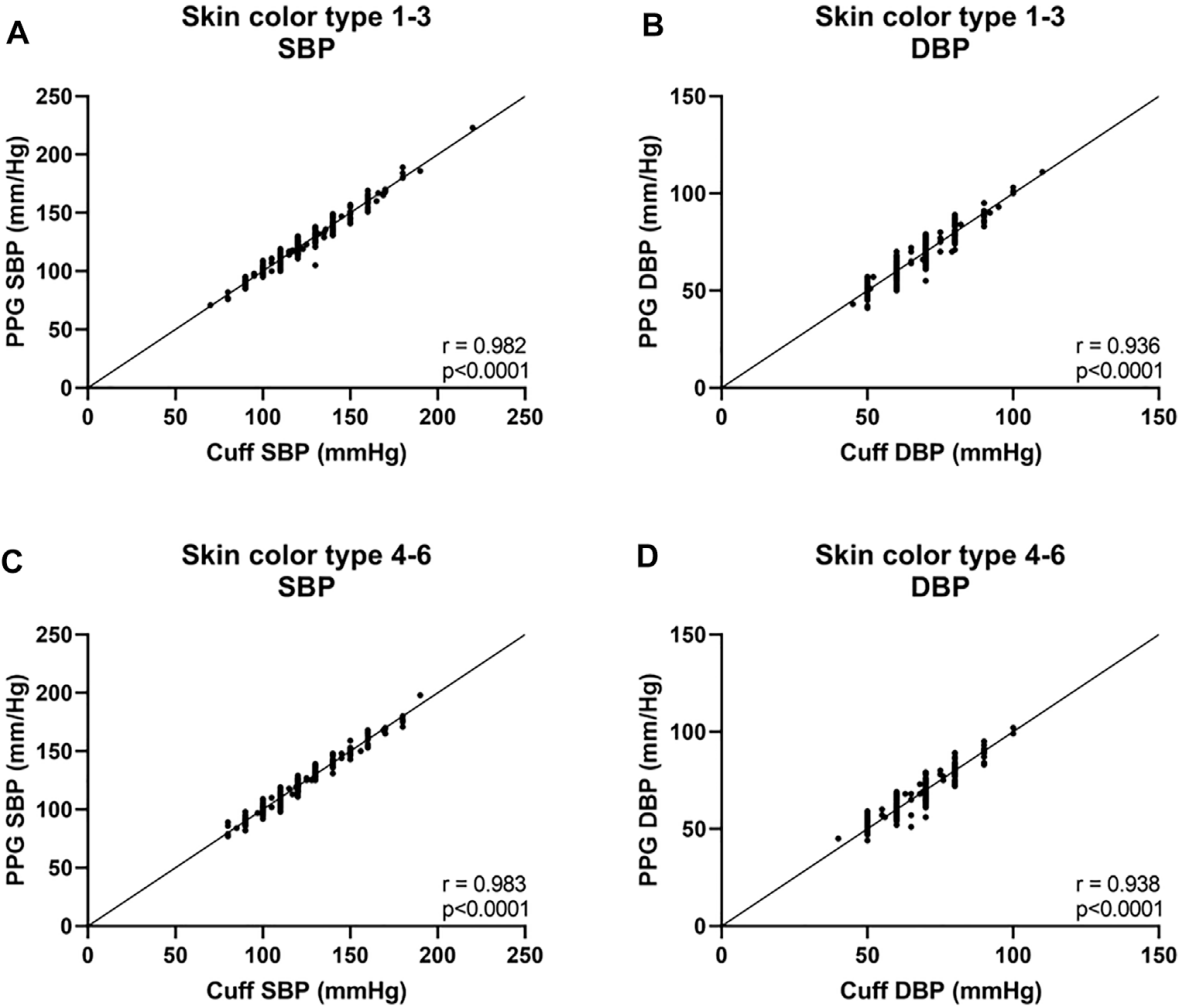
Correlation curves of the body’s skin color are based on the Fitzpatrick color scale (Fitzpatrick, 1988). Type 1—always burns, never tans, palest, can have freckles; Type 2—usually burns, tans minimally, light-colored but darker than fair; Type 3—sometimes mild burn, tans uniformly, golden honey or olive; Type 4—burns minimally, always tans well, moderate brown; Type 5—very rarely burns, tans very easily, dark brown; and Type 6—never burns, deeply pigmented dark brown to darkest brown. SBP systolic blood pressure, DBP diastolic blood pressure.

Next, we analyzed the data of the two groups defined by the Fitzpatrick scale. For SBP, the Bland-Altman analysis showed that the PPG has a bias of −0.1 ± 3.9 mmHg with 7.4 and −7.4 mmHg 95% LOA in type 1–3 group, r = 0.982; and −0.1 ± 3.8 mmHg with 7.2 and −7.5 mmHg 95% LOA in type 4–6 group, r = 0.983 (Figures 5A,C and Figures 6A,C). For DBP, the Bland-Altman analysis showed that the PPG has a bias of −0.4 ± 3.8 mmHg with 7.1 and −7.9 mmHg 95% LOA in type 1–3 group, r = 0.936; and −0.6 ± 3.9 mmHg with 7.1 and −8.3 mmHg 95% LOA in type 4–6 group, r = 0.938 (Figures 5B,D and Figures 6B,D).

We also tested the level of agreement between the PPG-based wearable measurements across commonly accepted standard (Table 2). Overall, we found that the level of agreement between divides for all sub-population was ~80% within 5 mmHg, 94% within 7 mmHg, and 99% in the range of 10 mmHg.

## Discussion

In this study, we found a high level of accuracy of BP obtained by cuffless PPG-based BP device as compared to cuff-based BP measurements, an agreement that was not influenced by sex, BMI, or skin color.

Hypertension is a leading risk factor for cardiovascular morbidity and mortality in industrialized and developing countries. Despite numerous developments in treatment, one-third of patients are considered to suffer from uncontrolled BP. The diagnosis and continuous management of BP rely on accurate BP measurements. Although BP measurements using sphygmomanometer are common and acceptable practice they suffer from detrimental limitations, including the provision of only point measurements, they are cumbersome, uncomfortable, and in many patients, the measurements are unreliable due to technical issues (Kronish et al., 2017; Ruzicka and Hiremath, 2017).

According to the WHO, in 2016, 39% of adults aged 18 years and over (39% of men and 40% of women) were overweight, and about 13% of the world’s adult population (11% of men and 15% of women) were obese (see URL in the Statistical analysis section). BMI and skin color are regarded as two important personal characteristics when looking at BP measurements, particularly as African Americans and obese are at higher risk of hypertension (Muntner et al., 2018; Chrysant, 2019; Ferdinand et al., 2020).

Novel PPG-based devices may provide continuous BP measurements with ease, making long-term BP monitoring and tailored therapy adjustments a reality (Nachman et al., 2021). Some PPG-based oximeters demonstrated unreliable measurements on people with darker skin tone (Ries et al., 1985; Ries et al., 1989; Jubran and Tobin, 1990; Zeballos and Weisman, 1991; Young, 1995). It is also accepted that cuff-based BP measurements are influenced by an individual’s BMI. Thus, these two personal characteristics could lead to biased and less accurate measurements and have an impact on the quality of care, and it is of utmost importance to assure accurate measurement when using a PPG-based device.

In the current study, we compared BP measurements using a novel PPG-based non-invasive cuffless BP device with a gold standard non-invasive cuff-based BP device, and when stratifying the participants based on sex, BMI, and skin color, we found a high correlation and accordance between BP measurements obtained using the two devices in all sub-groups. We have previously shown that the device stands for the ANSI/AAMI/ISO 81060-2 requirements, for intermittent BP measurement, as defined in Stergiou et al., 2018 (Nachman et al., 2020a).

The PPG-based device may allow transformative monitoring options with substantial clinical impacts such as improving BP control and diagnosis. Our group demonstrated the accuracy of the PPG-based device for continuous 24-h BP measurement in a small pilot study and validation studies are now undertaken by our group to show the accordance with other physiological parameters recorded using the same PPG-based devices, including more advanced hemodynamic parameters such as stroke volume, cardiac output, and systemic vascular resistance.

Future studies in a large population in which several BP measurements will be taken for each participant along several time points, rather than single time points, could further strengthen the observations of this study.

A limitation of this study is that we did not follow an accepted standard such as the ANSI/AAMI/ISO 81060-2 (Stergiou et al., 2018), however the standard deals with cuff-based devices, and there are currently no accepted standards beyond unofficial recommendations regarding validation of cuff-less devices. Moreover, this was a field study in which we did not want to interfere too much with the routine. For this reason, we had only one evaluator per patient measuring the BP and assessing the skin type color, and not two. This also allowed us to collect data from more people in a relatively short period and a real-world setting.

## Conclusion

We have found that the PPG-based BP device provides valid measurements regardless of sex, skin tone, or BMI. These findings provide confidence in the generalizability of such technology, helping in paving the way to a future of seamless, personalized BP management.

## Reporting Summary

Further information on research design is available in the Nature Research Reporting Summary linked to this article.

## Data Availability

The raw data supporting the conclusion of this article will be made available by the authors, without undue reservation.

## References

[B1] Adler J. N. Hughes L. A. Vtvilecchia R. Camargo C. A. Jr. (1998). Effect of Skin Pigmentation on Pulse Oximetry Accuracy in the Emergency Department. Acad. Emerg. Med. 5, 965–970. 10.1111/j.1553-2712.1998.tb02772.x 9862586

[B2] Bickler P. E. Feiner J. R. Severinghaus J. W. (2005). Effects of Skin Pigmentation on Pulse Oximeter Accuracy at Low Saturation. Anesthesiology 102, 715–719. 10.1097/00000542-200504000-00004 15791098

[B3] Bothma P. A. Joynt G. M. Lipman J. Hon H. Mathala B. Scribante J. (1996). Accuracy of Pulse Oximetry in Pigmented Patients. S. Afr. Med. J. 86, 594–596. 8914569

[B4] Chrysant S. G. (2019). Pathophysiology and Treatment of Obesity‐Related Hypertension. J. Clin. Hypertens. 21, 555–559. 10.1111/jch.13518 PMC8030569 30907058

[B5] Feiner J. R. Severinghaus J. W. Bickler P. E. (2007). Dark Skin Decreases the Accuracy of Pulse Oximeters at Low Oxygen Saturation: The Effects of Oximeter Probe Type and Gender. Anesth. Analgesia 105, S18–S23. 10.1213/01.ane.0000285988.35174.d9 18048893

[B6] Ferdinand D. P. Nedunchezhian S. Ferdinand K. C. (2020). Hypertension in African Americans: Advances in Community Outreach and Public Health Approaches. Prog. Cardiovasc. Dis. 63, 40–45. 10.1016/j.pcad.2019.12.005 31863786

[B7] Fitzpatrick T. B. (1988). The Validity and Practicality of Sun-Reactive Skin Types I Through VI. Arch. Dermatol. 124, 869–871. 10.1001/archderm.1988.01670060015008 3377516

[B8] Jubran A. Tobin M. J. (1990). Reliability of Pulse Oximetry in Titrating Supplemental Oxygen Therapy in Ventilator-Dependent Patients. Chest 97, 1420–1425. 10.1378/chest.97.6.1420 2347228

[B9] Kronish I. M. Kent S. Moise N. Shimbo D. Safford M. M. Kynerd R. E. (2017). Barriers to Conducting Ambulatory and Home Blood Pressure Monitoring During Hypertension Screening in the United States. J. Am. Soc. Hypertens. 11, 573–580. 10.1016/j.jash.2017.06.012 28734798 PMC5595651

[B10] Miller V. M. (2014). Why Are Sex and Gender Important to Basic Physiology and Translational and Individualized Medicine? Am. J. Physiol. Heart. Circ. Physiol. 306, H781–H788. 10.1152/ajpheart.00994.2013 24414073 PMC3949049

[B11] Muntner P. Carey R. M. Gidding S. Jones D. W. Taler S. J. Wright J. T. (2018). Potential US Population Impact of the 2017 ACC/AHA High Blood Pressure Guideline. J. Am. Coll. Cardiol. 71, 109–118. 10.1016/j.jacc.2017.10.073 29146532 PMC5873591

[B12] Nachman D. Constantini K. Poris G. Wagnert-Avraham L. Gertz S. D. Littman R. (2020b). Wireless, Non-Invasive, Wearable Device for Continuous Remote Monitoring of Hemodynamic Parameters in a Swine Model of Controlled Hemorrhagic Shock. Sci. Rep. 10, 17684. 10.1038/s41598-020-74686-6 33077774 PMC7573605

[B13] Nachman D. Gepner Y. Goldstein N. Kabakov E. Ishay A. B. Littman R. (2020a). Comparing Blood Pressure Measurements Between a Photoplethysmography-Based and a Standard Cuff-Based Manometry Device. Sci. Rep. 10, 16116. 10.1038/s41598-020-73172-3 32999400 PMC7527983

[B14] Nachman D. Gilan A. Goldstein N. Constantini K. Littman R. Eisenkraft A. (2021). Twenty-Four-Hour Ambulatory Blood Pressure Measurement Using a Novel Non-Invasive, Cuff-Less, Wireless Device. Am. J. Hypertens. 34, 1171–1180. 10.1093/ajh/hpab095 34143867

[B15] O’Brien E. (1996). Review: A Century of Confusion: Which Bladder for Accurate Blood Pressure Measurement? J. Hum. Hypertens. 10, 565–572. 8953199

[B16] Palatini P. Benetti E. Fania C. Saladini F. (2019). Only Troncoconical Cuffs Can Provide Accurate Blood Pressure Measurements in People with Severe Obesity. J. Hypertens. 37, 37–41. 10.1097/hjh.0000000000001823 29927843

[B17] Palatini P. Parati G. (2011). Blood Pressure Measurement in Very Obese Patients: A Challenging Problem. J. Hypertens. 29, 425–429. 10.1097/hjh.0b013e3283435b65 21317721

[B18] Pipek L. Z. Nascimento R. F. V. Acencio M. M. P. Teixeira L. R. (2021). Comparison of SpO2 and Heart Rate Values on Apple Watch and Conventional Commercial Oximeters Devices in Patients with Lung Disease. Sci. Rep. 11, 18901. 10.1038/s41598-021-98453-3 34556765 PMC8460792

[B19] Ries A. L. Farrow J. T. Clausen J. L. (1985). Accuracy of Two Ear Oximeters at Rest and During Exercise in Pulmonary Patients. Am. Rev. Respir. Dis. 132, 685–689. 10.1164/arrd.1985.132.3.685 4037542

[B20] Ries A. L. Prewitt L. M. Johnson J. J. (1989). Skin Color and Ear Oximetry. Chest 96, 287–290. 10.1378/chest.96.2.287 2752811

[B21] Ruzicka M. Hiremath S. (2017). Accuracy-Limiting Factor of Home Blood Pressure Monitors? Am. J. Hypertens. 30, 661–664. 10.1093/ajh/hpx056 28430845

[B22] Sjoding M. W. Dickson R. P. Iwashyna T. J. Gay S. E. Valley T. S. (2020). Racial Bias in Pulse Oximetry Measurement. N. Engl. J. Med. 383, 2477–2478. 10.1056/nejmc2029240 33326721 PMC7808260

[B23] Stergiou G. S. Alpert B. Mieke S. Asmar R. Atkins N. Eckert S. (2018). A Universal Standard for the Validation of Blood Pressure Measuring Devices: Association for the Advancement of Medical Instrumentation/European Society of Hypertension/International Organization for Standardization (AAMI/ESH/ISO) Collaboration Statement. J. Hypertens. 36, 472–478. 10.1097/hjh.0000000000001634 29384983 PMC5796427

[B24] US Food and Drug Administration (2013). Pulse Oximeters - Premarket Notification Submissions [510(k)s]: Guidance for Industry and Food and Drug Administration Staff. Available at: https://www.fda.gov/regulatory-information/search-fda-guidance-documents/pulse-oximeters-premarket-notification-submissions-510ksguidance-industry-and-food-and-drug (Last Accessed on 11/25/2021).

[B25] Wiles M. D. El‐Nayal A. Elton G. Malaj M. Winterbottom J. Gillies C. (2021). The Effect of Patient Ethnicity on the Accuracy of Peripheral Pulse Oximetry in Patients with COVID‐19 Pneumonitis: A Single‐Centre, Retrospective Analysis. Anaesthesia 77, 143–152. 10.1111/anae.15581 34542168 PMC8653100

[B26] Young K. (1995). A Study of the Accuracy of Pulse Oximetry Among Varying Skin Pigmentation Populations. Am. J. Crit. Care. 4, 256.

[B27] Zeballos R. J. Weisman I. M. (1991). Reliability of Noninvasive Oximetry in Black Subjects During Exercise and Hypoxia. Am. Rev. Respir. Dis. 144, 1240–1244. 10.1164/ajrccm/144.6.1240 1741533

